# Evaluation of antimicrobial resistance, biofilm forming potential, and the presence of biofilm-related genes among clinical isolates of *Pseudomonas aeruginosa*

**DOI:** 10.1186/s13104-020-4890-z

**Published:** 2020-01-10

**Authors:** Esmat Kamali, Ailar Jamali, Abdollah Ardebili, Freshteh Ezadi, Alireza Mohebbi

**Affiliations:** 1 Laboratory Sciences Research Center, Golestan University of Medical Sciences, Gorgan, Iran; 2 Department of Microbiology, Faculty of Medicine, Golestan University of Medical Sciences, Gorgan, Iran; 3 Stem Cell Research Center, Golestan University of Medical Sciences, Gorgan, Iran

**Keywords:** Antimicrobial resistance, Biofilm formation, Biofilm genes, *Pseudomonas aeruginosa*

## Abstract

**Objectives:**

*Pseudomonas aeruginosa* is known as a leading cause of nosocomial infections worldwide. Antimicrobial resistance and biofilm production, as two main virulence factors of *P. aeruginosa*, are responsible for the persistence of prolonged infections. In this study, antimicrobial susceptibility pattern and phenotypic and genotypic characteristics of biofilm of *P. aeruginosa* were investigated.

**Results:**

A total of 80 clinical *P. aeruginosa* isolates were obtained. Isolates showed resistance to all antibiotics with a rate from 12.5% (n = 10) against amikacin and piperacillin/tazobactam to 23.75% (n = 19) to levofloxacin. Multidrug-resistant *P. aeruginosa* accounted for 20% (n = 16). 83.75% (n = 67) of isolates showed biofilm phenotype. All three biofilm-related genes were found simultaneously in 87.5% (n = 70) of *P. aeruginosa* and 13.5% (n = 10) of the isolates had none of the genes tested. From the results of the present study, combination therapy including an anti-pseudomonal beta-lactam (piperacillin/tazobactam or ceftazidime) and an aminoglycoside or carbapenems (imipenem, meropenem) with fluoroquinolones in conjunction with an aminoglycoside can be used against *Pseudomonas* infections. However, reasonable antimicrobial use and high standards of infection prevention and control are essential to prevent further development of antimicrobial resistance. Combination strategies based on the proper anti-pseudomonal antibiotics along with anti-biofilm agents can also be selected to eradicate biofilm-associated infections.

## Introduction

*Pseudomonas aeruginosa*, as one of the most common hospital pathogens, is involved in a wide of severe opportunistic infections, particularly in immunocompromised patients [1]. As a global problem, the increasing rate of multidrug-resistance (MDR) strains has resulted in the medical therapy against *P. aeruginosa* be complicated [2–4]. In addition, the ability of *P. aeruginosa* to produce biofilm is thought to be a main factor involved in chronic infections. Biofilms are complex of microbial cells embedded in an extracellular matrix composed of proteins, extracellular DNA, and exopolysaccharides, providing a protective life-style for bacteria and are extremely challenging and costly to treat by antimicrobial compounds [5].

The biofilm components of *P. aeruginosa* are composed of at least three distinct exopolysaccharides, including alginate, Psl and Pel [6]. Alginate is mainly produced by *P. aeruginosa* clinical isolates originated from the lungs of cystic fibrosis (CF) patients [7]. It is a linear polymer consisting of β-d-mannuronic acid and α-l-guluronic acid and has an important role in structural stability and protection of biofilm [8]. Alginate synthesis in *P. aeruginosa* is controlled by the *algACD* operon. AlgD encoded by *algD* is a GDP-mannose dehydrogenase that catalyzes the production of GDP-mannuronic acid from GDP-mannose [9]. The *algD* gene mediates the control of alginate biosynthesis and transcription of the Alg proteins, and also is responsible for the final production of precursor GDP-mannuronic acid, the foundation molecule for polymerization and alginate synthesis [7].

*P. aeruginosa* isolates obtained from environments often produce two different exopolysaccharides. Psl (polysaccharide synthesis locus) is a neutral polysaccharide composed of a repeating pentasaccharide, consisting of d-mannose, d-glucose, and l-rhamnose. Psl has been shown that provides cell–cell and cell–surface interactions during biofilm formation, thereby playing an important in the initiation of biofilm formation and protection of biofilm structure [10]. The *psl* operon consisting of 15 co-transcribed genes (*pslA* to *pslO*) is required for Psl synthesis. PslD protein, encoded by *pslD* gene, is localized in the periplasm/outer membrane and is required for biofilm formation, probably by the export of a biofilm-relevant exopolysaccharide [11]. The formation of a layer of polymer/cells at the air–liquid interface of a *P. aeruginosa* standing culture is termed pellicle formation that is controlled by the *pel* (pellicle) operon [12]. Pel is a cellulose-sensitive exopolysaccharide composed of 1→4 linked partially acetylated galactosamine and glucosamine sugars [13]. The *pel* operon is composed of seven genes (*pelA* to *pelG*) [14]. PelF has been suggested to be a soluble glycosyltransferase using UDP-glucose as a donor substrate toward the Pel exopolysaccharide biosynthesis [12].

Given the potential of biofilm in increasing antimicrobial resistance and, as a result, the persistence of infections caused by *P. aeruginosa*, identification of the isolates possessing such factor will help us to better understand the pathogenesis of the organism. This study aimed to evaluate the antimicrobial susceptibility pattern as well as the phenotypic and genotypic characteristics of biofilm in *P. aeruginosa* isolates.

## Main text

### Methods

Bacterial isolates were collected during September 2017 to August 2018 from clinical specimens of patients in three university affiliated hospitals in Gorgran, Iran. Laboratory identification of *P. aeruginosa* isolates were performed by standard microbiological and biochemical methods, including pigment production in agar, oxidase and catalase tests, reactions in triple sugar iron (TSI) agar, SIM (sulfide, indole, motility), and oxidative-fermentative (OF) media (Merck, Darmstadt, Germany), and finally, growth at 42 °C [2].

Susceptibility of isolates to different antibiotics was determined by disk diffusion agar method on cation-adjusted Mueller–Hinton agar (Merck, Darmstadt, Germany) according to the Clinical and Laboratory Standards Institute (CLSI) recommendations [15]. Antibiotic disks (MAST Diagnostics, Merseyside, UK) tested were ceftazidime (CAZ, 30 μg), piperacillin/tazobactam (PTZ, 100 μg/10 μg), ciprofloxacin (CIP, 5 μg), levofloxacin (LEV, 5 μg), gentamicin (GM, 10 μg), amikacin (AK, 30 μg), tobramycin (TOB, 10 μg), imipenem (IMI, 10 μg), and meropenem (MEM, 10 μg). *Escherichia coli* ATCC 25922 was used as a control for susceptibility testing. Multidrug-resistant *P. aeruginosa* (MDR-PA) was defined as isolate resistant to more than one antimicrobial agent in three or more antimicrobial categories [16].

Quantitative assessment of biofilm formation was performed by the colorimetric microtiter plate assay as described previously by Stepanović et al. [17] with some modifications. An overnight culture of *P. aeruginosa* was adjusted to the turbidity of a 1 McFarland standard. Suspensions were diluted 1:100 in 200 μL tryptic soy broth (TSB) containing 1% glucose (Merck, Darmstadt, Germany), and were then transferred into the sterile flat-bottomed 96-well polystyrene microplates (JET Biofil, Guangzhou, China). Wells were gently washed three times with sterile phosphate buffered saline (PBS, pH 7.3) after 24 h of incubation at 37 °C. Adherent biofilms were fixed by 99% methanol for 15 min, the solutions were removed, and the plate was air-dried. Biofilms were stained by 200 μL of crystal violet 0.1% (Sigma Chemical Co., St Louis, MO, USA) for 5 min at room temperature, and then rinsed by water and allowed to dry. Biofilm in each well was destained by treatment with 200 μL of 95% ethanol for 30 min. The optical density (OD) was measured at 570 nm using a microtiter plate reader (BioTek, Bad Friedrichshall, Germany). All experiments were performed in triplicate and repeated three times. In addition, a cut-off value (ODc) was established. It is defined as three standard deviations (SD) above the mean OD of the negative control: Odc = average OD of negative control + (3 × SD of negative control). The isolates were classified into the four following categories based upon the OD: non-biofilm producer (OD < ODc); weak-biofilm producer (ODc < OD < 2 × ODc); moderate-biofilm producer (2 × ODc < OD < 4 × ODc); strong-biofilm producer (4 × ODc < OD).

All *P. aeruginosa* isolates were evaluated for three biofilm-encoding genes, *algD*, *pslD*, and *pelF* by polymerase chain reaction (PCR) method, using specific primers [8] synthesized by Metabion company (Metabion international AG, Germany). DNA extraction was performed from bacterial colonies by boiling method. All three genes were amplified under the following thermal conditions: initial denaturation at 95 °C for 5 min, followed by 30 cycles of denaturation at 94 °C for 30 s, annealing at 60 °C for 40 s, extension at 72 °C for 40 s, and a final elongation step at 72 °C for 5 min. PCR products were analyzed with UV light after running at 100 V for 1 h on a 1% agarose gel stained with DNA safe stain (SinaClon, Tehran, Iran).

Chi squared test was performed on the relationship between categorical variables, including biofilm characteristics and antimicrobial resistance using SPSS software, 18.0 (SPSS Inc., Chicago, IL, USA). A *p* value < 0.05 was considered as statistically significant.

## Results

A total of 80 distinct *P. aeruginosa* isolates were obtained from patients, of which 44 (55%) were from males and 36 (45%) were from females. Analysis of *P. aeruginosa* distribution in clinical specimens indicated that the most isolates (n = 29, 36.25%) were originated from endotracheal secretions, followed by urine (n = 26, 32.5%), blood (n = 11, 13.75%), wound (n = 8, 10%), CSF (n = 4, 5%), and ear (n = 2, 2.5%).

**Fig. 1:**
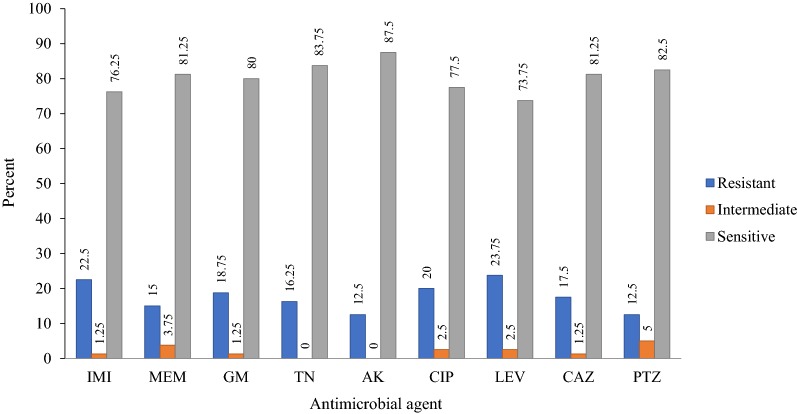
Antibiotic susceptibility patterns of *P. aeruginosa* clinical isolates. *IMI* imipenem, *MEM* meropenem, *GM* gentamicin, *TOB* tobramycin, *AK* amikacin, *CIP* ciprofloxacin, *LEV* levofloxacin, *CAZ* ceftazidime, *PTZ* piperacillin/tazobactam

Based on the CLSI interpretive criteria [15], resistance rate among *P. aeruginosa* isolates to antibiotics tested was as follow (Fig. 1): IMI 22.5% (n = 18), MEM 15% (n = 12), GM 18.75% (n = 15), TN 16.25% (n = 13), AK 12.5% (n = 10), CIP 20% (n = 16), LEV 23.75 (n = 19), CAZ 17.5% (n = 14), and PTZ 12.5% (n = 10). The prevalence of MDR-PA and non-MDR-PA was 20% (n = 16) and 80% (n = 64), respectively.

**Fig. 2:**
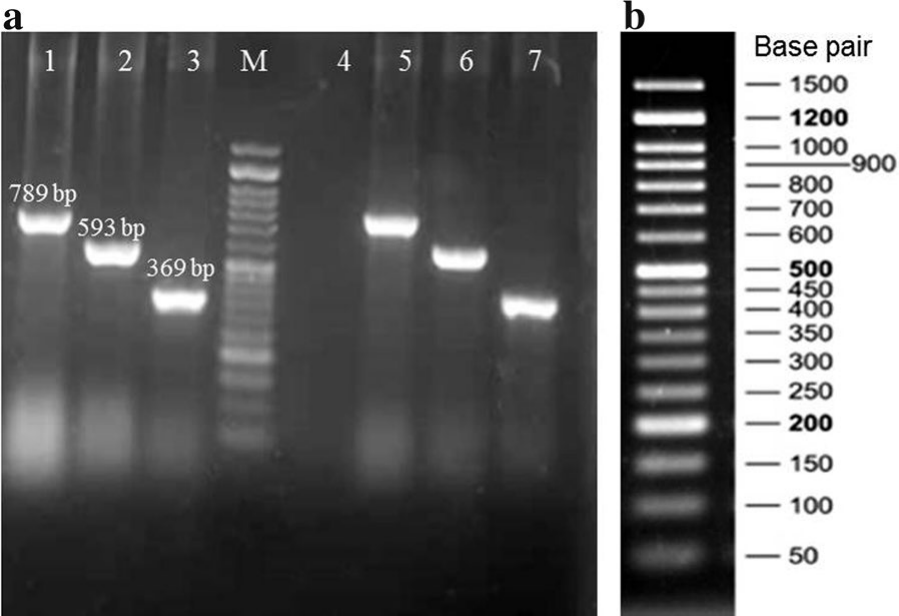
PCR amplification of biofilm-encoding genes in one selected clinical isolate of *P. aeruginosa* as representative. **a** Lane 1–3: PCR products of the *pelF*, *algD*, and *pslD* genes, respectively. M: 50 bp DNA ladder. Lane 4: PCR mixture without DNA template as control negative. Lane 5–7: PCR products of the corresponding genes in *P. aeruginosa* PAO1 reference strain as control positive. **b** A 50 bp DNA ladder containing seventeen discrete fragments ranging from 50 to 1500 bp with double intensity reference bands at 200 bp, 500 bp, and 1200 bp

Biofilm phenotypes accounted for 83.75% (n = 67), being distributed in the following categories: 16.25% (n = 13) produced strong biofilm; 33.75% (n = 27) produced moderate biofilm; 33.75% (n = 27) produced weak biofilm, whilst 16.25% of isolates (n = 13) were identified as non-biofilm producer (Table 1). A high occurrence of biofilm-encoding genes was found (Fig. 2): 87.5% (n = 70) of the isolates presented all three *algD*, *pslD*, and *pelF* genes, simultaneously (identified as *algD *^+^/*pslD *^+^/*pelF *^+^ genotypic pattern), while 12.5% (n = 10) had none and considered as *algD *^−^/*pslD *^−^/*pelF *^−^ pattern. In addition, isolates were divided to four groups based on both phenotypic and genotypic characteristics of biofilm: biofilm positive/gene positive (n = 59, 73.75%); biofilm negative/gene positive (n = 11, 13.75%); biofilm positive/gene negative (n = 8, 10%); biofilm negative/gene negative (n = 2, 2.5%). MDR phenotype accounted for 17.91% (n = 12) of 67 biofilm producers and 20% (n = 14) of 70 genotypically positive isolates.

**Table 1:** Relationship between biofilm characteristic and antibiotic susceptibility pattern among *P. aeruginosa* clinical isolates

Phenotypic pattern of biofilm, no. (%)	Genotypic pattern of biofilm, no. (%)		
	*AlgD *^+^/*pslD *^+^*/pelF *^+^	*AlgD *^−^/*pslD *^−^/*pelF *^−^	p-value
Strong, 13 (16.25)	10 (76.92)	3 (23.07)	0.001
Moderate, 27 (33.75)	25 (92.59)	2 (7.41)	0.0001
Weak 27 (33.75)	24 (88.89)	3 (11.11)	0.0001
Non-biofilm, 13 (16.25)	11 (84.61)	2 (15.38)	0.0001
Total, 80 (100)	70 (87.5)	10 (12.5)	0.0001

## Discussion

Development of resistance by *P. aeruginosa* to many antimicrobial agents is a great challenge in controlling its infections [1, 18, 19]. Comprehensive surveillance of antimicrobial resistance in European countries for 2017 demonstrated a range of combined resistance (resistance to three or more antimicrobial groups, including piperacillin ± tazobactam, ceftazidime, fluoroquinolones, aminoglycosides and carbapenems) from 0% (Iceland) to 59.1% (Romania) [20]. The prevalence of MDR *P. aeruginosa* in Iran has been estimated at 58%, with a variation in geographical areas: the highest and lowest rates were observed in Tehran (100%) and Zahedan (16%), respectively [21]. In a recent study, Bavasheh et al. [22] found that 27.8% of clinical *P. aeruginosa* isolates were MDR. Similarly, the rate of isolates with resistance to at least three antimicrobial groups in our study was 20% that was lower than that reported from other studies [8, 23, 24]. Although the rate of multi-resistance in the present study was relatively low, this may be an alarming situation that reflects a threat limiting treatment options in therapeutic centers studied.

Similar to the results of other studies [25, 26], a significant number of included isolates (83.75%) formed biofilm. The present study revealed a high prevalence of *algD*, *pslD*, and *pelF* genes, being presented simultaneously in a considerable proportion (87.5%) of *P. aeruginosa* isolates, a finding that are similar to those found by Banar et al. [8]. Other genes associated with biofilm formation, such as *pslA* and *pelA* were detected by Ghadaksaz et al. [27] with a frequency of 83.7% and 45.2%, respectively, and Pournajaf et al. [25] with a frequency of 89.5% and 57.3%, respectively among *P. aeruginosa* clinical isolates. However, little data is available about the prevalence rate of *pslD* and *pelF* genes in different regions of the world.

In agreement with other studies [8, 27], our results revealed a significant correlation between the biofilm forming capacity and the presence of relevant genes (p-value < 0.0001). About 88.06% of 67 biofilm producer isolates showed *algD *^+^/*pslD *^+^/*pelF *^+^ genotypic pattern, while 11.94% were *algD *^−^/*pslD *^−^/*pelF *^−^. On the other hand, 84.61% of 13 non-biofilm producers carried biofilm genes. The capacity of biofilm production despite the absence of biofilm genes studied indicates other genetic determinants of biofilm participate in matrix formation in *P. aeruginosa* [14, 28, 29]. By contrast, the presence of genes without biofilm production may be due to chromosomal mutations in different regulatory systems, affecting the production of functional biofilm-associated proteins. Hou et al. [30] reported that no *P. aeruginosa* isolates were phenotypically positive for biofilm formation in Congo red agar and microtiter plate assays, whereas 31.03% of isolates contained the *pslA* gene. Conformational changes in quorum sensing proteins due to mutations in *lasI/lasR* and *rhlI/rhlR* systems were suggested in previous studies [31–33] as the reason why these isolates are unable to produce biofilm.

According to the results of this study, *P. aeruginosa* that produced biofilm and also those carried biofilm-associated genes were mainly considered as non-MDR. This may cause a misunderstanding in the first place that biofilm production is not related to antibiotic resistance. It is noteworthy that all isolates in our study were subjected to antimicrobial susceptibility testing as planktonic cells and not in biofilm form. Thus, multiple mechanisms of biofilm and its architectural features, including glycocalyx matrix, outer membrane structure, heterogeneity in metabolism and growth rate, persister cells formation, genetic adaptation, stress responses as well as quorum sensing conferring MDR phenotype were not involved [34]. Regardless, Lima et al. [31] in Brazil found 48.4% of biofilm producer *P. aeruginosa* isolates were MDR and 51.6% were non-MDR. In another study, Abidi et al. [35] reported that biofilm production was significantly higher in MDR isolates.

In conclusion, combination therapy including an anti-pseudomonal beta-lactam (piperacillin/tazobactam or ceftazidime) and an aminoglycoside or carbapenems (imipenem, meropenem) with fluoroquinolones in conjunction with an aminoglycoside can be used against *Pseudomonas* infections. Although the rate of resistance to multiple antibiotics among the *P. aeruginosa* isolates was relatively low in the present study, prudent antimicrobial use and high standards of infection prevention and control are essential to prevent further development of resistant strains. In addition, combination strategies based on the proper anti-pseudomonal antibiotics with anti-biofilm agents can be used to enhance the treatment of biofilm-associated infections.

## Limitations

This study may be limited by the lack of clinical information of the patients (treatment, prescription drugs, mortality rate, length of a hospital stay) from whom bacteria were isolated. Furthermore, this study indicates that determination of expression levels of biofilm-associated genes by quantitative real-time PCR may help to evaluate the role of each corresponding gene in biofilm production.

## Data Availability

The data associated with this study are available from the corresponding author on reasonable request.

## Abbreviations

CF: cystic fibrosis
CLSI: Clinical and Laboratory Standards Institute
MDR-PA: multidrug-resistant *P. aeruginosa*
OD: optical density
OF: oxidative-fermentative
Pel: pellicle
PCR: polymerase chain reaction
Psl: polysaccharide synthesis locus
SIM: sulfide, indole, motility
TSB: tryptic soy broth
TSI: triple sugar iron
